# Collectanea

**Published:** 1830-02

**Authors:** 


### COLLECTANEA.

Floriferis ut apes in saltibus omnia libant,
Omnia nos, itidem, depascimur aurea dicta.

### PATHOLOGY.

*Tubercles found in the Lungs of a Child aged two months.* By Dr. BRICHETEAU. *(Journal Complementaire, &c.)*

THE mother of this infant was of a very weakly constitution, the father had had several attacks of pulmonary disease, and the grandfather and aunt had died of consumption. The child was born with an inguinal hernia on the right side, and the various symptoms under which it laboured during its short existence were attributed to inflammation of the intestines. It was pale and emaciated; had constant diarrhœa, short and laborious breathing, and occasional cough. At the age of two months the child died. The body was examined by Dr. B. and M. ELEMUNCEAU. The lungs on both sides were thickly studded with miliary tubercles. The right lung was hard and compact, and externally of a red colour, and contained throughout its whole extent numerous miliary granulations. It appeared impossible that this lung could permit the free transmission of air. About three fourths of the left lung were in the same state. It was externally redder than the right. Heart and pleuræ natural. The intestines were very pale, and presented no appearance of disease. The abdominal ring, through which the hernia had descended, was sufficiently dilated to admit a finger.

In the opinion of M. Bricheteau, the tuberculous affection of the lungs had not developed itself after birth. He concluded it to be a hereditary transmission from the father and grandfather. This fact is not devoid of interest, inasmuch as many pathologists assert that, although the *disposition* to tubercular consumption may be transmitted by parents to their offspring, the tubercles are always formed after birth, and rarely at a very early period of life.

*Case of Quotidian Remittent Fever, caused by an Abscess near the Anus, and treated ineffectually by Quinine.* By Dr. BRICHETEAU. *(Ibid.)*

A lady had suffered many days from a paroxysm of fever, with shivering, heat, and sweating. The type was evidently not intermittent; for, after the paroxysm, the pulse still indicated the continuance of febrile action, and there remained headach, anorexia, and wandering pains in the limbs. After having ascertained that there was no visceral disease, Dr. B. prescribed six grains of sulphate of quinine, after which the paroxysm did not occur in so short a period. The second dose, of nine grains, produced great irritation of the stomach, and the attack of fever returned at the same hour as at first.

From the failure of so powerful a remedy, the patient was more closely questioned, and it was ascertained that she was troubled with piles, which had caused her much suffering, but that she had not mentioned this circumstance, as her mind was entirely occupied by her febrile disease. Upon examination, Dr. B. found not only hemorrhoidal tumors, but a phlegmonous swelling, about the size of a pigeon's egg, situated immediately below the coccyx. This tumor was hard, painful on pressure, and evidently contained matter. Leeches were applied, and afterwards an emollient poultice; mild diet. The abscess shortly opened into the rectum, and a considerable quantity of pus was discharged. A second opening took place externally, near the margin of the anus, by which pus was also discharged, and a part of the clysters that were administered. A fistula, consecutive to the abscess, had formed. The paroxysms of fever ceased from the day the abscess broke. The patient was much relieved, and was ultimately entirely cured by the operation for fistula in ano.

The candid statement of this case is highly creditable to the relater of it. Dr. B. confesses the erroneous diagnosis he originally formed, and trusts that his open avowal may prevent similar mistakes, by impressing upon practitioners the urgent necessity of accurately examining their patients at their first visit, when a definite opinion is too often formed from insufficient inquiries.

---

*Pulmonary Tubercles.* In the October Number of the Ephemerides Medicales, 1828, is a very interesting analysis of an essay upon Tubercles, by M. Henri Clermond Lombard, of Geneva. According to this author, tubercles are an inorganic secretion, and augment in size by juxtaposition. He distinguishes them into simple and compound. The first are isolated, and the different molecules of which they are composed are united without any intermediate substance; the second result from the agglomeration of several simple tubercles: these, originally separated, in approaching each other, enclose in their intervals a portion of the organ in which they are developed, the life of which is destroyed by the tubercle thus formed.

Contrary to the opinion of Laennec and Louis, M. Lombard maintains that what have been termed pulmonary granulations are not tubercles in their commencing state, but a variety of chronic pneumonia, a hypertrophy of the vesicular parietes, as Andral had already described them to be. They may also, M. Lombard is convinced, be the result of a hypertrophy of the external cellular coat of the pulmonary vessels.

According to Laennec, Louis, and others, tubercles become softened from the centre to the circumference. This opinion is combated by the present author: according to him, the softening is produced by the action of the surrounding living structure, in its attempt to free itself from this morbid secretion, which, as a foreign body, impedes its functions. The molecules of the tubercle are separated from each other, and softened by the pus secreted around them: in this state of fluidity they are readily expelled. The softening, therefore, according to M. Lombard, takes place from the circumference towards the centre of the tubercle.

What is the seat of tubercles? Are they developed in the cellular tissue which unites the different portions of an organ; or do they form upon the surface of the mucous membranes? Bayle, Baillie, and Blainville consider the first opinion as demonstrated; Laennec and Louis think it probable that they may be developed upon mucous surfaces, while, according to Cruveilhier, this is undoubtedly the case. M. Lombard is of opinion that no facts have as yet occurred to demonstrate the latter supposition: he conceives it very probable that tubercles are developed in the cellular tissue, and that, if they have been found in the different canals, or in the cavities of the body, this has been in consequence of erosion or ulceration having permitted them to be removed from the part in which they were originally deposited. That tubercles are not formed, as some have supposed, within the bronchia or air-cells of the lungs, would appear to be proved by the observations of Gendrin, who ascertained, by injections and microscopical examinations, that the bronchia were free and permeable, notwithstanding the existence of tubercles in the lungs: it is thus he distinguishes them from the morbid muco-purulent secretion, which fills the final air-cells in severe cases of bronchial catarrh, and which many physicians have confounded with tubercles.

With respect to the connexion between inflammation and tubercles, whether it is sufficient for their production, or (as some maintain) has no effect whatever upon them, M. Lombard, taking the medium between these two extremes, is of opinion that it acts only as their occasional cause.

According to M. Lombard, the remote causes of tubercles are an hereditary predisposition, the lymphatic temperament, the sanguineo-nervous temperament, a cold and humid state of the atmosphere, inhabiting unhealthy situations, youth, the female sex, and an alteration of the fluids arising from bad food. The occasional are, sanguine or passive congestions, the activity of one or more organs, and the alteration of the fluids.

---

### PRACTICAL MEDICINE.

*Application of Iodine to Scrofula.* The following is part of a report by MM. MAGENDIE, SERRES, and DUMERIL, on a Memoir by M. LUGOL.

"M. le Dr. Lugol has treated 109 scrofulous cases, at the Hôpital Saint Louis, within seventeen months, with iodine alone. At the end of the last year, 39 still remained under superintendence, 30 had left the hospital very much improved, 36 had gone away completely cured, and in 4 cases only did the remedy seem quite inefficacious. The author concludes, from this mass of experience, that iodine should be considered as the most powerful and efficacious remedy in scrofulous cases, since it has constantly arrested the progress of the disease, or at least has exerted a salutary action upon the tuberculous tumors, where it has not decidedly produced their cure; and therefore that, in that point of view only, its introduction into medicine is one of the most valuable acquisitions the healing art has made in late times." The reporters say they are able to bear witness to the curative power of iodine in these cases, and consider the memoir and exertions of Dr. Lugol as highly useful.—*Revue Encyclop.*

---

### SURGERY.

*Laryngotomy.* A man, aged twenty-five years, was irritating his nostril with a needle, when, by some accident, he suffered it to enter the nostril, through which it passed, and fell into the pharynx. The needle was armed with a large thread, which disappeared entirely. Much irritation and cough were excited, by which the thread of the needle were thrown out. The patient endeavoured, by means of the thread, to extract the needle, but in vain. The respiration and voice becoming affected, and renewed attempts at extraction failing, the patient entered the Hôpital Beaujon, on the 18th of June, 1828. The symptoms of inflammation, swelling, &c. in the pharynx and larynx were now considerable; the voice was nearly lost; spasmodic contractions of the muscles of the neck were excited, and the soft parts in front of the trachea were much swollen.

The house student, having in vain endeavoured to extract the needle by means of the thread, sent for the surgeon, M. Blandin. The thread had now re-entered the pharynx, and all researches by means of the finger and forceps would not enable him to determine where the needle was fixed, whether in the larynx or pharynx. As the difficulty of respiration was still supportable, no operation was undertaken, but antiphlogistics, general and local, were employed with some advantage. On the evening of the 21st, the thread was again ejected, by means of which M. Blandin discovered that the needle had entered the superior aperture of the larynx on the left of the epiglottis. On the 22d the operation of laryngotomy was performed, by making a cautious dissection through the indurated and swollen parts, in front of the larynx, then carefully puncturing the crico-thyroid membrane, and afterwards dividing, by means of a director and bistoury, the thyroid cartilage, through its whole length on the median line. The respiration was now much relieved, and an attempt was made to discover and remove the needle by means of the forceps; but the irritation excited was so great as to induce the operator to desist. The wound was lightly dressed by means of a perforated compress covered with simple cerate, and the patient removed to bed. The night was passed comfortably; and the next day the needle, of a black or bronzed colour, and nineteen lines in length, was found fixed in the compress covering the wound.

The wound gradually healed, so that by the beginning of September merely a small fistula remained; but the voice was hoarse, there was some pain at the larynx, and other indications of chronic inflammation, for which leeches, a seton, &c. were prescribed with little advantage. The treatment was, however, continued, caustic was applied to the fistula, and by the 30th of September the sinus was closed, and the voice acquired more force.—*Rev. Med.*

---

*Wound of the Heart.* Dr. Leonard Randall, of Tennessee, relates the case of a negro boy, shot in the breast with a common fowlingpiece, loaded with shot, who lived for two months and six days after the accident. On dissection, it was found that the right lung was nearly destroyed, the left lung was much inflamed, and several shot were found in its substance; the pericardium was partially adherent, and part was absorbed; while the heart was enlarged, partially adherent to the pericardium, and its parietes indurated. On opening the right ventricle, three shot were found in its cavity! This ventricle was greatly enlarged, and lined with a thick coat, from which there projected numerous papillæ of a dun colour, giving it the appearance of the upper surface of the tongue of an ox. Two shot were also found in the right auricle, but the internal surface was not much injured by their presence.—*Western Journal of Med. and Phys. Sciences.*

---

### MIDWIFERY.

*Labour retarded by a preternatural Septum in the Vagina.* Professor Richardson, of the Transylvania University, relates the case of a female in labour with her third child, in whom delivery was prevented by a preternatural septum in the vagina. After a careful examination, the head was found presenting and pressing against the new membrane, which however yielded very little to vigorous uterine contractions. A small orifice was eventually discovered in the centre of the septum, through which the liquor amnii had been discharged. Dr. R. divided this septum, by making a small incision with a probe-pointed bistoury, and then enlarging the opening with the finger towards the rectum and pubis. Labour advanced rapidly, although some resistance was still made by the lips of the septum. Mother and child did well, although the latter was born in a state of asphyxia.

From the history given by the patient, there was no satisfactory explanation of the occurrence of this preternatural membrane. Her second labour, although tedious and painful, was not followed by any bad consequences, and she was not conscious of having ulcerations or any other disease of the vagina. —*Trans. Journal of Med.*

---

*Ossification in the Placenta.* In the Vierteljährigen Sanitätsberichten, (Hufeland and Osann's Journal, June 1828,) Dr. KATERBAU states that, in the month of February, 1828, a woman was delivered of a healthy female child. She had complained, many weeks previous to her confinement, of a pain in the womb, and a feeling as if something within it pricked and cut her: for these sensations many remedies were administered without effect. After the tolerably speedy birth of the child, the placenta did not come away, and the midwife, supposing it to adhere, proceeded to loosen it, which was easily done. During the operation, the patient complained of violent pricking within the uterus.

Upon inspecting the placenta, remarks the Doctor, I found that throughout its substance were interspersed numerous spicula of bone, the whole of which resembled the points of ossification in a fœtal skull. They were firmly united to the integuments of the placenta, and in some parts, especially over the insertion of the cord, were arranged together so as to present somewhat of an arborescent appearance.

---

*On the Means of affording Respiration to Children in reversed Presentations.* By JACOB BIGELOW, M.D. Professor of Materia Medica in Harvard University. (Extracted from the *American Journal of Med. Sciences.*)

It is familiar to obstetric practitioners, and is noticed by most writers on Midwifery, that in those cases of labour in which the body of the child is delivered before the head, a considerable degree of danger exists in regard to the life of the child. Rules for the conduct of such cases are laid down by writers, yet it cannot be denied that, in the hands even of skilful practitioners, many children, which are alive when the body is expelled, are irrecoverably lost before the head can be extracted. In these cases, death takes place because the connexion with the mother is interrupted, by compression of the cord, or detachment of the placenta, before a communication with the atmosphere is effected.

It is the object of the present paper to show, that in many such cases the life of the child may be saved, by forming a communication between the mouth and the atmosphere, previous to the delivery of the head.

After the body is expelled, if the head can be seasonably delivered, either by the recurrence of pains or by the successful efforts of the practitioner, no difficulty ordinarily occurs. But this desirable state of things cannot always be realized. Too frequently the size of the head, and the resistance of the pelvis or soft parts, renders the delivery difficult and hazardous; and the practitioner, in the midst of his efforts, is apprised by a convulsive jerk or spring of the body, that a state of extreme danger exists, and that the time has come at which the child must breathe or will speedily die. If at this period the fingers be introduced, so as to reach the mouth of the child, it will be perceived that each jerk of the body is attended with a gasp and convulsive effort at inspiration, performed by the mouth and chest of the child. In this state of things, if air be conveyed to the mouth of the child, it will immediately breathe, and the efforts of nature, as will hereafter be shown, may in most cases be safely waited for to assist in expelling the head.

The method to be pursued in conveying air to the mouth, depends upon the situation of the head. If the chin has descended low in the pelvis, so that the mouth rests upon the perineum or lower part of the sacrum, and can be readily reached by the fingers, the hand of the operator alone is sufficient to give the assistance required. But if the mouth is situated so high in the pelvis as to be reached with difficulty, or if, from the relative size of the head, there is much compression, the assistance of a tube may be of use. The mode of proceeding which I have found successful in various instances, is as follows: As soon as the body and arms are extracted, supposing the face towards the sacrum, an assistant supports the body, carrying it towards the pubis; or the reverse, should the position of the face be to the pubis. The accoucheur should then introduce the hand to which the face looks, till the middle fingers rest upon the mouth of the child. The hand is then to be raised from the throat of the child, making the ends of the fingers a fulcrum, and pushing the perineum backwards. The air will thus pass upwards as far as the chin of the child. The middle fingers are now to be separated about half an inch from each other, and thus a complete passage will be formed between them, by which the air will reach the mouth of the child. If the child be in a healthy state up to this period, it will immediately breathe and cry, and the delivery of the head may be safely postponed until the natural pains recur. If, from any degree of asphyxia, the child does not immediately breathe, it may often be made to do so by dashing cold water upon the body, or by any other stimulating processes. It has even appeared to me practicable to inflate the lungs in some cases, through an elastic catheter. When the mouth is so high in the pelvis as to be reached with difficulty, or when the compression is so great as to obliterate the cavity between the fingers, a flat tube will be found useful, made of metal, of spiral wire covered with leather, or of elastic gum, and having its largest diameter about half an inch. If the tube be of metal, or of any incompressible material, it should be withdrawn during a pain, to prevent contusion of the soft parts, and immediately replaced, if the pain subsides without expelling the head. Such a tube may be considered as a prolongation of the trachea, and is fully sufficient to sustain life by respiration for a considerable time. The tube must be guarded and directed by keeping it between the fingers of the inserted hand.

The following are a part of the cases which have occurred to me in practice, affording an opportunity for the trial of this method.

CASE I. A patient was in labour with her second child, August 8th, 1824. The case was one of breech presentation, and without any unusual occurrence the body and arms were delivered in about three hours after my arrival. The position of the head was of the most common kind, with the vertex above the pubis, and the face in the lower part of the hollow of the sacrum. At this time my left hand was passed upward, with a view of depressing the chin, but the child being large, it required some effort to reach the mouth with the fingers. The time consumed in doing this was too great for the safety of the child, and the convulsive spring of the body took place. I was forcibly struck at the same moment by perceiving a gasp of the mouth at the ends of my fingers, and the idea occurred that, if a communication could be made to the atmosphere, the child would respire. Attempts were made, without success, to extract the head by a moderate force, aided by the efforts of the mother and by pressure made by an assistant over the fundus of the womb. At the same time the hand which rested over the mouth and throat was raised a little, and the fingers opened to give passage to the air. The child soon gave another convulsive spring, and at the same moment inspired. The hand being retained in the same position, a slow but constant respiration continued, accompanied with a low, moaning cry for eight or ten minutes, when the recurrence of a pain caused the head to be delivered. During the whole of this period, before the final pain, the mouth was several inches within the perineum.

Case II. This case occurred May 1st, 1826, and was also a breech case. Being a first labour, it was protracted for eighteen hours. After the presentation was ascertained, I had made, in a hasty manner, a tube about five inches long and half an inch wide, slightly flattened, and slightly bent over at its extremity. The case being one of more than common interest, I provided myself likewise with forceps. Although the mother had been in perfect health, yet the body of the child, when expelled, was found emaciated and dark-coloured, exhibiting marks of feeble life. As much force as it was thought justifiable to use was employed to extract the heap, with no other effect than to bring the mouth within about two inches of the edge of the perineum. The tube was now introduced, and placed in the mouth of the child, but it did not respire. It will be observed, that the child had exhibited no convulsive effort, nor any signs of being alive. An attempt was now made to inflate the lungs, which failed, apparently from want of tightness in the tube, the joining not having been soldered. It nevertheless appeared to me practicable to have inflated the lungs in this situation, with a suitable tube, since the tightness with which the perineum covers the face would assist in preventing regurgitation of the air. The foregoing attempts having proved unavailing, the forceps were introduced, with the aid of which the head was extracted. The child was resuscitated with great difficulty, and did not breathe spontaneously until artificial respiration had been kept up, by inflating the lungs through a quill, for more than half an hour. It was two hours before the respiration became so perfect that the child could be left to itself. I have no doubt that this child would have respired before the birth of the head, had there been sufficient constitutional vigour to produce the effort.

---

### CHEMISTRY.

*New Source of Spirit.* It is stated that the berries of the Sorbus Aucuparia are now used in the north of France for the production of a spirit, and the result is said to be equal to the purest distillation from grapes for brandy. The berries, when perfectly ripe, are first exposed to the action of cold in the open air, then put into a wooden vessel, bruised, and boiling water poured on; the whole being stirred until it has sunk in temperature to 82° F. A proper quantity of yeast is then added, the whole covered up, and left to ferment. When the fermentation is over, the liquor is to be put into the still and drawn over in the usual way. The first running is weak and disagreeable in flavor, but, being distilled from off very fresh finely powdered charcoal, in the proportion of eight or nine pounds to forty gallons of weak spirit, a very fine product is obtained. The charcoal should remain in the liquid two or three days before the second distillation.

---

*Passage of Iodine into the Blood.* M. BENNERSCHEIDT has published, in the Archiv. des Apotheker, a notice of his examination of the blood of an individual who had for a long time employed frictions with iodine ointment. The serum gave no indication of the presence of the iodine, but it was detected in the crassamentum by a slight blue tinge which it communicated to starch.—*Journal de Chimie Médicale.*

---

### NATURAL HISTORY.

*Curious Phenomenon in Vegetable Physiology.* M. ALIX, of St. Valery, near Somme, has in his possession an apple-tree, the source of which is unknown, and the age supposed to be forty years. This tree, which perfectly resembles the ordinary apple-tree in its leaves and the disposition of its flowers, differs by the flowers being deficient of petals and stamina, but possessing instead fourteen styles and a calyx with ten segments, connected below, but disposed in two alternate ranges. The peduncle of the flower is woolly, and the styles, being slightly hairy at the base, are surmounted by a very viscid oblique stigma. In consequence of the organization of these flowers, the tree was sterile, until, it having been suggested that artificial fecundation could be effected by means of pollen taken from other apple-trees, the tree was made to produce fruit. Since then it has become a sort of festival in the country to render the tree productive; and those of the neighbourhood who feel an interest in the progress of the tree, when it comes into flower, so soon as they meet with a perfect apple flower elsewhere in fine dry weather, they pluck it, and apply it to one of the flowers of the sterile tree, and leave it there until it falls off; then the person distinguishes the flower by attaching a coloured ribbon to it, so that in the autumn each may know his apple.

The apples differ much from each other in their size, taste, and colour, because of the variety in the trees from which the perfect flowers were taken; but they are all distinguished by a degree of contraction, situated nearly about one third from the end. Within each apple are fourteen cells, situated in two horizontal and parallel planes; five of these are disposed as in the ordinary apple, in the middle of the fruit; but the other nine, which are smaller, are nearer to the top of the apple. Each cell does not always contain a seed; the number of the latter varies from three to nine. The arrangement of these cells has some analogy with the appearance which two apples would present if fastened end to end, and of which the longitudinal section would present the figure of a leaf in the shape of a violin or panduriform.

WILDENOW, POIRET, and others, have described unisexual apple-trees, in which the petals and stamina were absent; but they differ from the tree of St. Valery in their fecundation being effected by the vicinity of other apple-trees, and in having only a five-leaved calyx, from five to ten styles, and five cells in the fruit. M. TILLETTE DE CLERMONT explains the present case by the theory of junctions and miscarriage developed by M. DE CANDOLLE. In applying this theory to the tree in question, it must be imagined that there is the flower of an ordinary apple-tree, from which are developed two other flowers, which, instead of being supported on separate peduncles, must be considered as joined together, and at the same time conjoined with the simple flower from which they have their origin; and this in such a manner, that the soldered ovaria of the two upper flowers are superposed and soldered to the ovarium of the inferior flower, a style and a cell being at the same time suppressed. This natural monstrosity, therefore, is the product of three flowers soldered together, in which there is at the same time suppression of the petals, stamina, a calyx, and a pistil. The examination of the fruit leaves no doubt on this subject, and evidently proves the truth of M. Clermont's hypothesis. —*Revue Encyclop.*

---

*On the Prickle which exists in the Tail of the Lion.* Two lions, which died some months ago in the menagerie of the King's Garden at Paris, have furnished an occasion of verifying a curious fact, mentioned in some old works, but which modern authors have generally omitted: it is that there exists at the extremity of the lion's tail a small claw, concealed in the midst of the tuft of long black hairs which occurs there. It is a horny production, about two lines in length, which presents itself under the form of a small cone a little curved, and adhering by its base to the skin only, and not to the last vertebra, which is separated from it by a space of two or three lines. This small claw exists in both sexes. The commentators of Homer thought they could explain, by the presence of this claw, a curious and correct remark made by the author of the Iliad, which was, that the lion is the only animal which, when irritated violently, agitates its tail, and strikes its sides with it. They imagined tha the lion sought to excite himself by pricking his sides with the horny production in question. Blumenbach, some years ago, verified the existence of this prickle; but the pamphlet in which his observations are contained has remained unnoticed by naturalists, and the curious fact of which we speak might long have remained unknown, had not M. Deshayes happened to see the pamphlet in question, and engage the naturalists who more particularly study the department of mammalogy, to make some observations on the subject. This prickle, or spur, adhering only to the skin by the circumference of its base, is very easily detached. In general, no traces of it remain in stuffed individuals. It has not yet been observed whether it exists equally in the other large species of the genus Felis.—*Edinb. New Philos. Journal.*

---

*On a dangerous Plant growing among Watercresses.* The procumbent water-parsnip, or Sium Nodiflorum, is a dangerous plant of the umbelliferous class, which grows mixed with watercresses in springs and streams: when not in flower, it so much resembles the latter, that it is with difficulty distinguished except by a botanist. Watercresses are of a deeper green, and sometimes spotted with brown, and the extremities of the leaves are more round, and especially the last leaves, which are in pairs larger than the others, and undulated at their edges. The water parsnip, on the contrary, is of an uniform green, the ends of its leaves are longer and narrower, conical at the extremities, and toothed at the edges. The best method of knowing them well, is to examine them in July, when their flowers are expanded, and when they may be thoroughly distinguished from each other.—*Quarterly Journal of Science.*

### MISCELLANEOUS.

*Air introduced into the Veins of a Cock.* M. Dieffenbach having procured a strong large cock, two years old, with a very thick and large comb, propelled into the jugular vein as much air as he was able at one continued expiration. The animal made a loud noise, and fell dead; its wings and thighs were strongly convulsed. In one second, the comb, which was before of a deep red colour, was spotted white and blue. The pulsation of the heart ceased immediately after the introduction of the air; the pupils were much dilated. When small portions of the comb were cut off, a quantity of frothy blood issued from the little wounds. Some of the bubbles adhered to the comb, and several of them acquired the size of a lentil, in consequence of air issuing from the vessels of the part. When the whole of the froth was wiped away, the same phenomenon again occurred, and continued for several hours. The next day the body of the bird was examined. The left cavities of the heart were nearly empty. The right were full of black coagulated blood. The large veins also full of coagulated, and the arteries of liquid blood. Air could not be detected in any part excepting here and there between the laminæ of mesentery, which were raised up like globular vesicles. The whole of the skin had a spongy feel, probably from air being contained in its minute vessels.—*Journal Complementaire.*

---

*Mechanical Leech.* A mechanic of Brussels has recently invented an ingenious instrument, intended to be used instead of leeches, when they are scarce, or cannot be procured. It consists of a triangular punch, which makes a wound exactly similar to the bite of a leech. Within this instrument is a small exhausting pump, composed of a sucker placed below the punch, and a little piston also furnished with a sucker. When the piston is raised, the sucker within the cylinder permits the blood drawn to ascend, and when the piston is pushed down, the lower sucker closes, and the blood is thrown from the instrument. By means of this ingenious little apparatus, as much blood may be drawn as is required, and it may be applied precisely upon any diseased part.—*Industriel Belge.*

